# Immune checkpoint inhibitors-related pancreatitis with fulminant type 1 diabetes mellitus: case report and literature review

**DOI:** 10.3389/fimmu.2023.1243773

**Published:** 2023-09-28

**Authors:** Wei Fang, Yang Gao, Xiaoyan Shi, Xiaoran Zhang, Shan Zhou, Hongxia Zhu, Wei Yan, Huanping Wang

**Affiliations:** ^1^ Department of Endocrinology, Chengdu Shuangliu Hospital of Traditional Chinese Medicine, Chengdu, China; ^2^ Department of Ultrasound, West China Hospital, Sichuan University, Chengdu, China; ^3^ Department of Human Cell Biology and Genetics, School of Medicine, Southern University of Science and Technology, Shenzhen, China; ^4^ Department of Endocrinology, Hospital of Chengdu University of Traditional Chinese Medicine, Chengdu, China

**Keywords:** immune checkpoint inhibitors, immune-related adverse events, immunotherapy, case report, literature review

## Abstract

Immune checkpoint inhibitors (ICIs) are increasingly being used in the treatment of advanced human malignancies. ICIs-related adverse events, including pancreatitis and diabetes, have been individually characterized in the literature. The co-occurrence of ICIs-related pancreatitis with diabetes is rare and easily overlooked, but it is often severe or fatal. We present a patient with renal tumor resection who was treated with injection of the PD-L1 inhibitor toripalimab and eventually developed acute pancreatitis and fulminant type 1 diabetes mellitus. In addition, we conducted a literature review of ICIs-related pancreatitis with diabetes. The case in our report presented with paroxysmal abdominal pain and loss of appetite. Intravenous fluids and insulin infusion improved the patient’s pancreatitis and explosive hyperglycemia. This article suggests that ICIs can affect endocrine and exocrine functions of the pancreas, while providing information and new perspectives for the diagnosis and treatment of this challenging rare disease, helping inspire clinicians for the early identification and effective management of similar cases.

## 1 Introduction

Immune checkpoint inhibitors (ICIs) include anticytotoxic T-lymphocyte-associated protein 4 (anti-CTLA-4), antiprogrammed cell death 1 receptor and its ligand (anti-PD-1/anti-PD-L1) (1). These novel drugs can enhance the immune system’s ability to eliminate cancer cells by triggering the reactivation and expansion of T lymphocytes (1, 2). Therefore, ICIs are widely used to treat malignant melanoma, non-small cell lung carcinoma (NSCLC), head and neck squamous cell carcinoma, and malignancies of the genitourinary and hematological systems (3). During the past decade, the life expectancy of end-stage cancer patients has increased significantly because of using ICIs (4).

However, the widespread use of ICIs has been followed by a series of immune-related adverse events (irAEs) associated with their mechanism of action. These irAEs can influence almost all organ systems, from minor self-limiting symptoms to severe life-threatening incidents, including the dermatologic, gastrointestinal, hepatic, pulmonary, and endocrine systems (5). ICIs-associated pancreatic injury (ICIs-PI) is rare but can cause metabolic and nutritional disorders, and even death (6, 7). Timely recognition of the condition and initiation of treatment can hugely impact the patient’s health and quality of life. In this paper, we described a patient with acute pancreatitis (AP) and fulminant type 1 diabetes mellitus (FT1DM) induced by toripalimab (anti-PD-1), conducted a systematic review of relevant case reports and attempted to raise awareness about ICIs-PI.

## 2 Case descriptions

A 58-year-old Chinese man (70.0 kg, body mass index: 21.1 kg/m^2^) diagnosed with clear cell renal cell carcinoma (WHO/ISUP nuclear grade was 4) underwent left nephrectomy in August 2021. In December 2021, the patient began immunotherapy with toripalimab (240 mg/dose), a humanized antiprogrammed cell death 1 receptor. Eleven weeks after initiating toripalimab treatment, he visited the hospital with a seven-day history of paroxysmal abdominal pain and the resulting loss of appetite. He had a 30-year history of smoking but had quit smoking for a year and had no history of alcohol consumption. Furthermore, he had no personal or family history of endocrine or autoimmune diseases.

### 2.1 Disease progression

**Figure 1:**
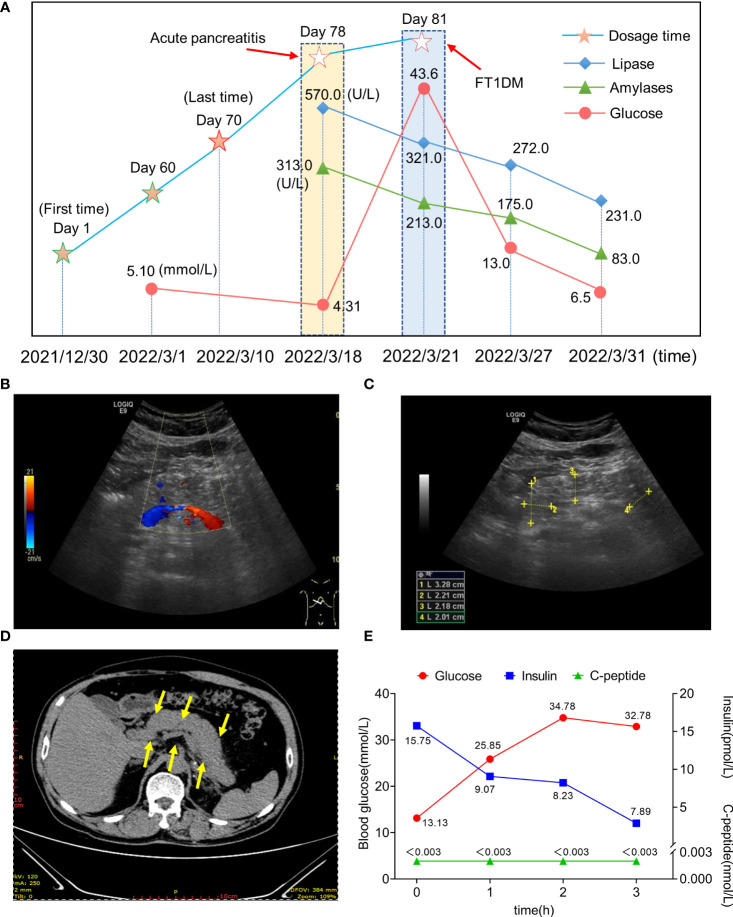
**(A)** The clinical course of irAEs. **(B, C)** Ultrasound showed a diffusely enlarged and hypoechoic pancreas. **(D)** The abdominal CT scan showed diffuse enlargement of the pancreas with mild surrounded by inflammatory stranding [yellow arrow]. **(E)** Experimental results of the release of the insulin and C-peptides.

**Figure 1A** shows the overall changes during the disease course of the patient. A day before admission, the patient’s mid-upper abdominal pain worsened. After admission, the patient’s blood amylase was 313 U/L (28.0-100.0 U/L), blood lipase was 670 U/L (23.0-300.0 U/L), and random blood glucose was 4.3 mmol/L. Total cholesterol and triglycerides were slightly elevated, while liver and kidney function, electrolytes, blood gas analysis and blood ketone bodies were not significantly abnormal. Glycosylated hemoglobin was 7.3%. Abdominal ultrasound revealed an “enlarged pancreas with uneven parenchymal echogenicity” (**Figures 1B, C**), and abdominal computed tomography (CT) showed a “full pancreas with slightly blurred peripheral space” (**Figure 1D**). On the third day after hospitalization, the patient developed dry mouth and excessive fluid intake, increased urination, and fatigue. Laboratory tests showed the patient’s blood glucose was 43.6 mmol/L, and urine glucose was positive (+++), while other indicators were not notable. After the patient’s condition stabilized, we performed oral glucose tolerance tests (OGTT), insulin, and C-peptide release experiments along with islet autoantibodies. Data showed the patient had a complete loss of islet function (**Figure 1E**) and was negative for islet autoantibodies (**Supplementary Table 1**). Subclinical hyperthyroidism (TSH 0.01 mIU/L, FT4 21.85 pmol/L) was also detected, and thyroid autoantibodies (TPOAb, TSI) were negative. The thyroid ultrasound did not show hypervascularization. Detailed laboratory test values for the patient are shown in **Supplementary Table 2**. We continuously tested the patient’s lipase and glucose levels during treatment. The patient’s symptoms subsided after two weeks of treatment, and his lipase levels returned to 231.0 U/L, amylase levels to 83.0 U/L, and fasting glucose to 6.5 mmol/L; Subsequently, the patient was discharged from the hospital.

### 2.2 Diagnostic assessment

After completing four immunotherapy cycles, the patient experienced abdominal pain and elevated levels of pancreatic enzymes. The patient had no gallstones, normal triglycerides, no personal history of autoimmune disorders, and no alcohol abuse or overeating. An abdominal CT examination revealed diffuse pancreatic enlargement with no signs of tumor metastasis, leading to the diagnosis of ICIs-related pancreatitis (ICIs-P) (8, 9). After three days of hospitalization, the patient developed symptoms of dry mouth and thirst, with a sudden increase in blood sugar to 43.6 mmol/L and complete loss of pancreatic islet function (C-Peptide <0.003). The patient had no history of diabetes (3 weeks before admission, FBG 5.1 mmol/L, HbA1c 6.1%) and only a slight increase in glycated hemoglobin (HbA1c 7.3%) on admission. Therefore, FT1DM was diagnosed (10). According to the Naranjo Adverse Drug Reaction Probability Scale, the total score was 6; therefore, we considered that pancreatic damage was associated with toripalimab therapy in this case (11). Patients with elevated blood sugar levels after acute pancreatitis must be differentiated from those with postacute pancreatitis diabetes mellitus (PPDM-A) and stress diabetes mellitus. Glucose metabolism in PPDM-A is like that in T2DM with surviving islet function. Stress hyperglycemia manifests itself mainly as insulin resistance leading to increased blood sugar, which can return to normal after the stress factor has been eliminated (12, 13). Therefore, PPDM-A and stress hyperglycemia were not considered in this patient, but a diagnosis of ICIs-FT1DM was considered. The patient had no previous episodes of thyrotoxicosis, while subclinical hyperthyroidism (TSH 0.001 mIU/L, FT4 21.85 pmol/L) and negative thyroid autoantibodies (TPOAb, TSI) were measured at admission. This clinical situation was indicative of ICIs-related thyroiditis (14, 15).

### 2.3 Therapeutic intervention

According to the Common Terminology Criteria for Adverse Events (CTCAE 5.0) and the National Comprehensive Cancer Network (NCCN) guidelines (16), the patient’s elevation in blood glucose was classified as life-threatening (grade 4); amylase or lipase levels of > 2.0-5.0 × ULN were rated grade 3; therefore, toripalimab needed to be discontinued. After diagnosing pancreatitis induced by toripalimab, the patient’s vital signs were monitored, and fluids were replaced. The patient’s symptoms were relieved after treatment. However, on the third day following hospitalization, the patient abruptly developed FT1DM. So he was administered appropriate intravenous fluids and an insulin infusion. Subsequently, he still required insulin therapy. Therefore, blood glucose was controlled by subcutaneous insulin injections. Due to delays in thyroid autoantibody detection results and limited experience in this area, a small dose of methimazole was also used to reduce thyrotoxic symptoms in patients.When the diagnosis was confirmed, methimazole was immediately discontinued and clinical observations were performed in accordance with treatment guidelines (17, 18). The patient was discharged when his symptoms improved after two weeks of treatment. We revisited the patient three months later, who was doing well with stable glycemic control,thyroid function back to normal and no abdominal pain, signs of pancreatic exocrine insufficiency, or tumor aggravation.

## 3 Discussion

The incidence of ICIs-related pancreatic irAEs (pancreatitis, hyperglycemia, elevated amylase/lipase, exocrine pancreatic insufficiency) is relatively low, with an incidence of 0.5%-4%, but can lead to poor quality of life and even be life-threatening (19). ICIs-P or ICIs-DM as the most important pancreatic exocrine or endocrine toxicity of irAEs has been reported separately in some studies (2, 20). However, their incidence and clinical characteristics are unclear. To our knowledge, literature reports are scarce on the incidence of ICIs-P combined with ICIs-DM. We reviewed literature on ICIs-P combined with ICIs-DM but found only three studies on ICIs-P and DM (21–23). **Table 1** summarizes our case and the three cases reported to develop ICIs-P and ICIs-DM during treatment with ICIs. All patients were male with a mean age of 60.5 (range 49-72) years and had no history of diabetes. All three patients had a history of nivolumab treatment, and here we report the first case of ICIs-P combined with ICIs-FT1DM in a patient with renal cancer during treatment with toripalimab. Male sex and PD-1/PD-L1 blockers, particularly nivolumab, may be risk factors for immune-related pancreatitis and diabetes.

**Table 1:** Our case and summary of reported immune checkpoint inhibitors associated with pancreatitis and diabetes mellitus.

	Our Case	Case 1 Lea Dehghani 2018 (21)	Case2 Mesut Yilmaz 2021 (22)	Case 3 Wataru Munakata 2016 (23)
Diagnosis	urothelial carcinoma	metastatic melanoma	mRCC (IIIc)	cHL
Age of onset (years)	58	63	49	72
Sex	Male	Male	Male	Male
Agent(ICIs)	Toripalimab	Nivolumab	Nivolumab	Nivolumab
Time to onset of pancreatitis after ICIs	11 weeks (4 cycles)	15 months	34 months (72 cycles)	6 cycles
Time to onset of DM after ICIs	11 weeks (4 cycles)	18 months	10 months (22 cycles)	6 cycles
Tumor response to ICIs therapy	Complete response	Complete response	Partial response	Partial response
Relevant History	center nephrectomy	None	center radical nephrectomy and retroperitoneal lymph node dissection	None
Symptoms	paroxysmal abdominal pain and concomitant loss of appetite	asymptomatic	nausea, vomiting, and abdominal pain	slight thirst, polyuria, and general fatigue
Lipase(U/L)	670	63	1587	80
Amylases (U/L)	313	NR	728	NR
CT/MRI findings	enlarged pancreas (CT)	peripancreatic fatty infiltration (CT)	diffuse enlargement of the whole pancreas (CT)	diffusely enlarged (MRI)
FDG PET/CT	None	18F-FDG uptake	NR	NR
Grade	G3	G2	G3	G1
BG level at onset of DM (mg/dl)	43.6 mmol/L	11 mmol/L	44.4 mmol/L	375 mg/dL (20.83mmol/L)
DKA	No	No	Yes	No
HbA1c (%) at onset of DM	7.3%	78 mmol/mol	10.9%	7.3%
C-peptide (ng/ml)	<0.003	0.41 nmol/L	NR	NR
Islet autoantibodies	negative	negative	NR	negative
IgG4	None	0.33g/L(0.04–0.86)	NR	NR
Treatment of pancreatitis	Rehydration therapy	Insulin and Pancreatic enzyme replacement therapy	Methylprednisolone (iv, 2mg/kg) [only]	Intensive insulin replacement therapy
Discontinued ICIs	No (PD)	TAC	No (PD)	Yes (TDR)
Other endocrine toxicity	Thyroiditis	Liver injury	None	None
Outcome	improved	improved	improved	improved

mRCC, metastatic renal cell carcinoma; cHL, classical Hodgkin lymphoma; TAC, Treatment already completed at the onset; TDR, temporarily discontinued, then restarted; PD, permanent discontinuation; NR, No report.

Of the three cases collected, one patient had pancreatitis and diabetes mellitus simultaneously, similar to our case (23), a case had acute pancreatitis 2 years after FT1DM (22), and a case had elevated blood glucose 3 months after the onset of autoimmune pancreatitis (type II) (21). This suggests that endocrine and exocrine damage to the pancreas can occur simultaneously or sequentially without a strict order of onset. Regarding the time of onset, ICIs-P and ICIs-FT1DM occurred in our case 11 weeks after starting ICIs treatment. The literature reports that early toxic pancreatic toxicity of ICIs may occur on day 1 of ICIs treatment (6) or late toxicity may occur after >1 year of therapy with ICIs (24) or even after the end of treatment (25). Delayed toxicity has been reported in other studies, which occurred 1 to 2 years following monotherapy (22). Therefore, ICIs-PI can appear at any time of treatment, and it is critical to consider the possibility of immune-associated pancreatitis/diabetes if there are any new symptoms of pancreatic injury (abdominal pain, elevated pancreatic enzymes, blood glucose, etc.) during ICIs treatment.

**Figure 2:**
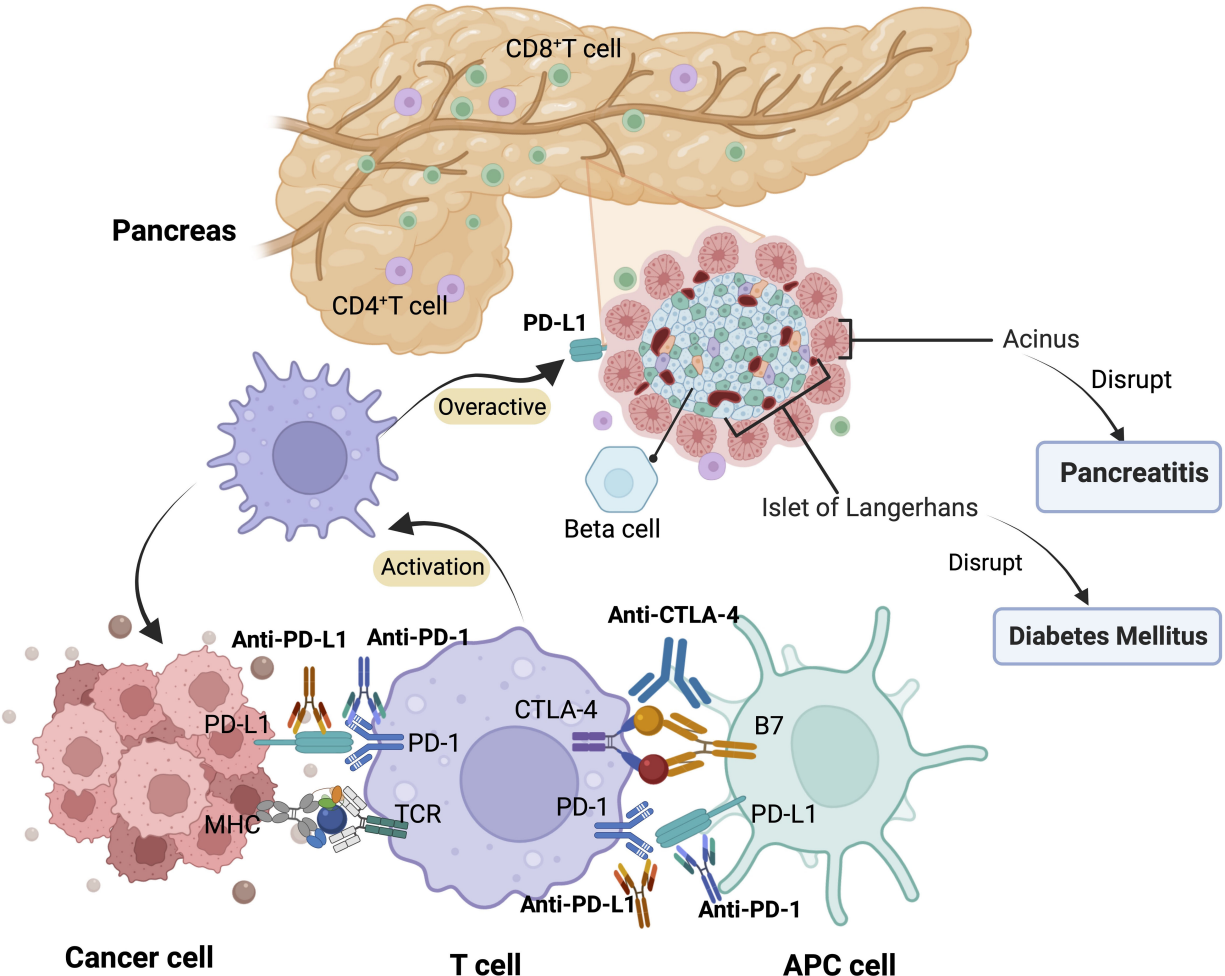
Mechanism of pancreatic adverse events related to ICIs. APC, antigen-presenting cell; MHC, major histocompatibility complex; TCR, T cell receptor.

The mechanism by which ICIs are associated with pancreatic injury remains unclear, although several factors may be involved. However, excessive activation of T-lymphocytes, leading to the destruction of pancreatic cells, may be an important part of the pathogenesis (5).PD-1 negatively regulates immunity; PD-1 on the surface of T cells binds to PD-L1 on the surface of tumor cells, and T cells receive inhibitory signals, inactivation, and apoptosis occurs. Anti-PD-1/PD-L1 and anti-CTLA-4 monoclonal antibodies bind and block inhibitory signals. Therefore, the inhibitory signal is lifted, and T cells regain their tumor-killing function (26). PD-L1 is expressed not only in T cells, but also in pancreatic cells. When the PD-1 pathway is blocked, cancer-targeted T cells are activated, increasing the ratio of CD8+/CD4+ T lymphocytes in the peritoneal area. Increased CD8 + T cells may cause damage to pancreatic cells, including islet and acinar cells, leading to decreased endocrine and exocrine pancreatic function (2). This explains why ICI-related pancreatic injury can present with endocrine and exocrine dysfunction of the pancreas (**Figure 2**). However, this does not explain the low incidence, diverse clinical episodes (hyperglycemia, pancreatitis, pancreatic atrophy, and pancreatic exocrine insufficiency), and significant heterogeneity (9, 25).The exact mechanism of immune-related pancreatitis/diabetes requires further research.

NCCN guidelines for the treatment of ICIs-P state that for grade 2 pancreatitis (moderate), ICI treatment should be discontinued and 0.5-1 mg/kg/day prednisone/methylprednisolone should be administered until symptoms improve to grade ≤ 1 (16). For pancreatitis (severe and life-threatening), it is recommended to permanently discontinue immunotherapy and start therapy with 1-2 mg/kg/day of glucocorticoids. When symptoms of ICIs-P have improved to grade 1, steroids can be gradually reduced over 4-6 weeks. However, the NCCN guidelines are based on traditional acute pancreatitis and limited evidence on ICI-P, so current treatment options may not be effective for managing ICI-P combined with new-onset diabetes. A review study showed that corticosteroids have not been shown to have a significant benefit in shortening the acute phase of ICIs-P, preventing long-term adverse outcomes of ICIs-P, or improving overall survival, while early intravenous fluids may reduce the risk of long-term adverse outcomes of ICIs-P (27). AP combined with FT1DM in this patient, using corticosteroids, may cause difficulty controlling blood glucose, which may lead to acute ketoacidosis. No corticosteroids were administered to our patient. However, he was managed with active rehydration and insulin hypoglycemic therapy. The patient’s pancreatic enzymes decreased, blood glucose control and symptoms improved, and no signs of exocrine damage to the pancreas were found during follow-up. This suggests the importance of early identification and intervention, even if basic therapy is effective (rehydration, hypoglycemic reduction, etc.). Based on published literature and our case experience, the use of glucocorticoids can be evaluated according to the following criteria when pancreatitis co-occurs with ICIs-related diabetes: 1. the severity grade of ICIs-P (Grade 3-4 pancreatitis necessitates consideration of steroid therapy); 2. whether pancreatitis and diabetes manifested simultaneously; 3. whether it was complicated by irAEs in other organs.”

## 4 Conclusions

Although the simultaneous occurrence of ICI-induced endocrine and exocrine pancreatic injuries is rare, it still requires more attention due to its life-threatening nature. Any new symptoms of pancreatic injury should be considered clinically significant for immunotherapy patients. Therefore, careful recognition and accurate diagnosis are extremely important. Despite the effectiveness of fluid therapy and insulin supplementation, the underlying mechanisms of immune pancreatic injury remain unclear and require further investigation.

## Data availability statement

The original contributions presented in the study are included in the article/**Supplementary Material**. Further inquiries can be directed to the corresponding authors.

## Ethics statement

The studies involving humans were approved by the Medical Ethics Committee of Chengdu Shuangliu Hospital of Traditional Chinese Medicine. The studies were conducted in accordance with the local legislation and institutional requirements. The participants provided their written informed consent to participate in this study. Written informed consent was obtained from the individual(s) for the publication of any potentially identifiable images or data included in this article. Written informed consent was obtained from the participant/patient(s) for the publication of this case report.

## Author contributions

WF, YG, and XS contributed equally to this work and share first authorship. YG and HW conceived and designed the study. WF were involved in clinical management. XZ, SZ, HZ, WY, and HW contributed to the literature search, data acquisition, and data analysis for the review. YG, WF, and XS drafted and revised the paper. YG contributed to all the figures. All authors contributed to the article and approved the submitted version.
