# Supplementary material for: Immune checkpoint inhibitors-related pancreatitis with fulminant type 1 diabetes mellitus: case report and literature review
Source: Front Immunol. 2023 Sep 28;14:1243773. doi: 10.3389/fimmu.2023.1243773 (PMC10569069; doi:10.3389/fimmu.2023.1243773)
Supplement: Supplementary file 1 [file DataSheet_1.docx]

Supplementary Material

Immune Checkpoint Inhibitors-Induced Pancreatitis withFulminant Type 1 Diabetes Mellitus: Case Report and Literature Review

Wei Fang^1†^, Yang Gao^2†*^, Xiaoyan Shi^3†^, Xiaoran Zhang^4^, Shan Zhou^4^, Hongxia Zhu^1^, Wei Yan^1^ , Huanping Wang^1*^

^1^Department of endocrinology, Chengdu Shuangliu Hospital of Traditional Chinese Medicine, Chengdu, China；

^2^Department of Ultrasound, West China Hospital, Sichuan University , Chengdu, China.

^3^Department of Human Cell Biology and Genetics, School of Medicine, Southern University of Science and Technology, Shenzhen, China.

^4^Department of Endocrinology, Hospital of Chengdu University of Traditional Chinese Medicine, Chengdu, China.

***Correspondence:**

Yang Gao, gaoyangxueshu@163.com. Huanping Wang, fangw139@163.com.

^†^These authors contributed equally to this work and share first authorship

**Keywords:** Immune checkpoint inhibitors, Immune-related adverse events, Immunotherapy, Case report, Literature Review

**Supplementary Table 1.** Results of islet autoantibodies.

| Index | Value | Reference |
| --- | --- | --- |
| Glutamic acid decarboxylase antibody（Anti-GAD） | Negative | （-） |
| Tyrosine phosphatase antibody（Anti-IA2） | Negative | （-） |
| Antibody to zinc transporter 8（Anti-ZnT8） | Negative | （-） |
| Anti-islet cell antibody（Anti-ICA） | Negative | （-） |
| Insulin antibodies（Anti-IA） | Negative | （-） |

**Supplementary Table 2** Laboratory data when ICIs-P and FT1DM were diagnosed

| Index | Value | | Reference |
| --- | --- | --- | --- |
|  | Diagnosis of ICIs-P  (2022.3.18) | Diagnosis of FT1DM  (2022.3.21) |  |
| Blood glucose (mmol/L) | 4.31 | 43.6 | 4.11–5.89 |
| Ketonemia (mmol/L) | - | <0.1 | <0.1 |
| Urea (mmol/L) | 6.6 | 6.1 | 2.14–7.14 |
| Creatinine (umol/L) | 97 | 102 | 59–104 |
| eGFR (mL/min/1.73m^2^) | >60 | >60 | >60 |
| HCO_3_ (mmol/L) | 21.3 | 20.7 | 22.0–29.0 |
| Na (mmol/L) | 138.5 | 134.3 | 136–145 |
| K (mmol/L) | 4.13 | 4.67 | 3.5–5.2 |
| Cl (mmol/L) | 98.8 | 93.6 | 96–108 |
| Ca (mmol/L) | 2.41 | 2.35 | 2.15–2.50 |
| Albumin (g/L) | 46.3 | 46.1 | 35–52 |
| AST (U/L) | 27.7 | 32.0 | 0.0–40.0 |
| ALT (U/L) | 40.0 | 41.0 | 0.0–42.0 |
| Total bilirubin (umol/L) | 15.8 | 7.3 | 2.5–21.0 |
| ALP (U/L) | 97.0 | 99.0 | 40.0–130.0 |
| TC (mmol/L) | 6.13 | 5.78 | 1.10–5.20 |
| TG (mmol/L) | 2.4 | 3.16 | 0.0–2.26 |
| Troponin T (pg/mL) | 32.71 | 21.67 | 0.0–14.0 |
| Serum Myoglobin (ng/mL) | 81.92 | 70.90 | 28–72 |
| Amylases (U/L) | 313 | 213 | 28.0–100.0 |
| Lipase (U/L) | 570 | 321 | 23.0–300.0 |
| White blood cell count (*10^9^/L) | 7.7 | 8.9 | 4.0–10.0 |
| Neutrophils (%) | 66.7 | 68.7 | 50.0–70.0 |
| Hemoglobin (g/L) | 146 | 148 | 130.0–175.0 |
| Platelet (*10^9^/L) | 172.4 | 150.0 | 100.0–300.0 |
| Blood gas analysis |  |  |  |
| pH | 7.35 | 7.403 | 7.33–7.42 |
| PaO_2_ (mmHg) | 82 | 86 | 65–80 |
| Base excess (mmol/L) | 1.5 | 1.26 | -2.0–3.0 |
| Lactate (mmol/L) | 1.2 | 3.2 | 0.7–2.1 |
| Urine |  |  |  |
| Urine Microalbumin (mg/L) | - | 11.2  +- | 0.0–25.0 |
| Protein | Negative | +- | - |
| Glucose | Negative | +++ | - |
| Urinary ketone | Negative | Negative | - |
| Thyroid |  |  |  |
| TSH (mIU/L) | - | 0.01 | 0.4–4 |
| FT4 (pmol/L) | - | 21.85 | 10.3–25.74 |
| TG-Ab (U/mL) | - | 0.77 | 0.0–4.11 |
| Thyroid ultrasonography | - | The right leaf has a strong echo of 2*2.4mm tuber | - |

**Abbreviations:** ICIs-P, ICIs-induced pancreatitis; FT1DM, fulminant type 1 diabetes mellitus; eGFR, estimated glomerular filtration rate; AST, aspartate aminotransferase; ALT, alanine aminotransferase; ALP, alkaline phosphatase; TC: total cholesterol; TG, triglyceride; TSH, thyroid stimulating hormone; FT4: free-thyroxine; TG-Ab, thyroglobulin antibodies; CRP, C-reactive protein.
